# Neoplastic transformation of porcine mammary epithelial cells *in vitro* and tumor formation *in vivo*

**DOI:** 10.1186/s12885-015-1572-7

**Published:** 2015-07-31

**Authors:** A. R. Rowson-Hodel, R. Manjarin, J. F. Trott, R. D. Cardiff, A. D. Borowsky, R. C. Hovey

**Affiliations:** 1 Department of Animal Science, University of California Davis, One Shields Avenue, Davis, CA 95616 USA; 2 Present Address: Department of Biochemistry and Molecular Medicine, University of California Davis School of Medicine, Sacramento, CA USA; 3 Present Address: USDA/ARS Children’s Nutrition Research Center, Baylor College of Medicine, Houston, TX USA; 4 Center for Comparative Medicine, University of California Davis, One Shields Avenue, Davis, CA USA

**Keywords:** Breast cancer model, Microenvironment, Lentivirus transformation, Xenograft, Pig

## Abstract

**Background:**

The mammary glands of pigs share many functional and morphological similarities with the breasts of humans, raising the potential of their utility for research into the mechanisms underlying normal mammary function and breast carcinogenesis. Here we sought to establish a model for the efficient manipulation and transformation of porcine mammary epithelial cells (pMEC) *in vitro* and tumor growth *in vivo*.

**Methods:**

We utilized a vector encoding the red florescent protein tdTomato to transduce populations of pMEC from Yorkshire –Hampshire crossbred female pigs *in vitro* and *in vivo*. Populations of primary pMEC were then separated by FACS using markers to distinguish epithelial cells (CD140a-) from stromal cells (CD140a+), with or without further enrichment for basal and luminal progenitor cells (CD49f+). These separated pMEC populations were transduced by lentivirus encoding murine polyomavirus T antigens (Tag) and tdTomato and engrafted to orthotopic or ectopic sites in immunodeficient NOD.Cg-*Prkdc*^*scid*^ *Il2rg*^*tm1Wjl*^/SzJ (NSG) mice.

**Results:**

We demonstrated that lentivirus effectively transduces pMEC *in vitro* and *in vivo*. We further established that lentivirus can be used for oncogenic-transformation of pMEC *ex vivo* for generating mammary tumors *in vivo*. Oncogenic transformation was confirmed *in vitro* by anchorage-independent growth, increased cell proliferation, and expression of CDKN2A, cyclin A2 and p53 alongside decreased phosphorylation of Rb. Moreover, Tag-transformed CD140a- and CD140a-CD49f + pMECs developed site-specific tumors of differing histopathologies *in vivo*.

**Conclusions:**

Herein we establish a model for the transduction and oncogenic transformation of pMEC. This is the first report describing a porcine model of mammary epithelial cell tumorigenesis that can be applied to the study of human breast cancers.

**Electronic supplementary material:**

The online version of this article (doi:10.1186/s12885-015-1572-7) contains supplementary material, which is available to authorized users.

## Background

Preclinical studies of breast cancer are limited by a lack of suitable models recapitulating aspects of human physiology and the biology of the human breast. Approximately 90 % of cancer treatments stemming from preclinical screens performed using xenografts in rodents fail during clinical trials [1], highlighting intrinsic genetic, physiological [2, 3] and morphological [4] differences between humans and mice. The pig offers a promising alternative to traditional rodent models given they share pronounced genomic [5] and biological [6] similarities to humans. As such, pigs have increasingly become an integral species for translational research, particularly for preclinical toxicology studies and as a biomedical model for human cardiovascular, integumentary and gastrointestinal systems [7].

While the mammary glands of female pigs have only been infrequently cited as a model for the human breast, they closely recapitulate several important aspects of human breast biology. Development of the mammary tissue in pigs from embryogenesis [8] through puberty [9] and gestation [10] parallels that of the human breast [11]. While pigs have an average of 10–14 mammary glands, each has multiple (2–4) galactophores that drain to the nipple and form the primary duct from which the parenchymal tissue develops [12]. Humans also have multiple galactophores per nipple, while the mouse has only one [13]. The histomorphology of the porcine mammary gland and human breast has been similarly described as having terminal ductal lobular units (TDLU) embedded within fibrous inter-and intralobular connective tissues [9, 11], which contrasts to the simple ductal network and adipose-rich stroma of the mouse mammary gland [4]. Importantly, intrinsic structural differences between the mammary glands of rodents and humans likely influence tumorigenic risk given that the stroma directs proliferative, morphogenic and hormonal responses by the epithelium [14–16]. Furthermore, the relative abundance of different TDLU morphotypes in the human breast can influence breast cancer risk, where the least-differentiated TDLU type 1 (TDLU-1) is most prone to transformation [17]. A porcine model of human breast cancer would stand to address many of these interactions that underlie breast development and tumorigenesis [18]. Moreover, the size and positioning of the voluminous mammary glands will allow for the assessment of multiple treatments or endpoints within an animal and over time using serial biopsies [19].

Further to the above, few reports detail methods to isolate and genetically manipulate the mammary epithelial cells (pMEC) in pigs. The objective of this study was to establish methods of lentivirus-mediated transformation of pMEC as a first step toward developing a novel model for human breast cancer. We hypothesized pMEC would undergo oncogene-induced transformation to yield tumors with a histopathology resembling human breast cancers. Herein, we report the successful lentiviral transduction of porcine mammary cells *in vitro* and tissue *in vivo*, formation of tumors by transformed pMEC in immunocompromised mice, and the precocious expansion of TDLU when transformed pMEC were isografted into the pig mammary gland.

## Methods

### Experimental design

We initially conducted experiments to determine the efficiency of using lentivirus for the transduction of pMEC *in vitro* and *in vivo*. In study one we sought to develop and optimize methods for the collection and dissociation of mammary tissue from nulliparous pigs for transduction *in vitro*. In study two we transduced pig mammary tissue *in vivo* by direct instillation of non-oncogenic lentivirus into the mammary gland duct or parenchyma. For study three, we sought to determine whether pMEC transduced with non-oncogenic lentivirus *in vitro* could develop typical mammary structures when transplanted back to the mammary fat pads of respective donor pigs. Finally, in studies four and five, pMEC were transformed *in vitro* by oncogenic lentivirus and either isografted to the mammary gland of donor pigs (study four) or xenografted to the mammary fat pad of immunocompromised mice (study five).

### Animals

All experimental protocols for animal experimentation underwent prior ethical review and were approved by the UC Davis Animal Care and Use Committee following guidelines set forth by the Association for Assessment and Accreditation of Laboratory Animal Care and the Guide for the Care and Use of Agricultural Animals in Research and Teaching (protocol #17675). For study one and study five, mammary tissue was obtained at necropsy from healthy nulliparous Yorkshire × Hampshire pigs obtained from the specific pathogen-free swine facility at UC Davis when they were 3–5 months of age (*n* = 8 and *n* = 4, respectively). For study two (*n* = 9 pigs from two litters), study three (*n* = 8 pigs from two litters), and study four (*n* = 4 pigs from two litters) pigs were healthy, experimentally naïve 4 week-old Yorkshire × Hampshire females. For studies two, three and four, piglets were selected that possessed at least twelve mammary glands, which permitted an individual pig to carry experimental treatments and controls within separate mammary glands. Piglets were housed indoors in a temperature-controlled facility (25–27 °C), as littermate pairs, were fed twice daily and had *ad libitum* access to water. Pigs were monitored daily for any changes in behavior or health status. During surgical procedures, pigs were assessed for changes in body temperature, heart rate and respiration. All surgical procedures involving pigs were carried out in a disinfected surgical suite designed for accommodating large animals.

In study five, 20 experimentally naïve female *NOD scid gamma* (NOD.Cg-*Prkdc*^*scid*^ *Il2rg*^*tm1Wjl*^/SzJ (NSG)) mice (The Jackson Laboratory, Sacramento, CA; *n* = 4 per pMEC line) between 4 and 35 weeks of age (Table 1) were maintained in littermate groups with *ad libitum* access to food and water. Mice were housed in a pathogen-free barrier facility under conditions of constant temperature (20–23 °C), humidity (45–65 %), and a 14 h light/10 h dark cycle. Tumor formation was assessed weekly by palpation, and tumor diameter recorded every 2d once they reached 1 mm diameter. During surgical procedures, mice were monitored for toe-pinch reflex and respiration rate. Surgical procedures were carried out within a disinfected biosafety cabinet to minimize pathogen exposure.

**Table 1:** pMEC were sorted to remove fibroblasts (CD140-), and some selected for expression of CD49f or tdTomato, and then transduced at various passages (P_T_) with one of the lentiviral constructs PGK-Tantigen (PGKT), CMV-tdTomato (CMVT) or PGKT-CMVT (PTCT)

Cell line	FACS	P_T_	*In vitro* morphology	Number	Age	P_I_	Cells (#)	Matrix	Site	E + P	Tumor diameter (mm)	Weeks carried	*in vivo* characteristics
*ss*071712 PGKT	CD140-	1	Cobblestone, no foci	3	67d	6	1×10^5^	Hydrogel	MG	No	N/a	16.7	N/a
*ss*071712 CMVT	CD140-					6	1×10^5^	Hydrogel	MG	No	N/a	16.7	
*ss*071712 PGKT	CD140-	1		7	74d	6	1×10^5^	Hydrogel	MG	No	N/a	15.7	N/a
*ss*071712 CMVT	CD140-					6	1×10^5^	Hydrogel	MG	No	N/a	15.7	
27-3 PTCT	CD140-tdTomato+	1	Foci, radial outgrowth	4	30d	7	5×10^5^	Hydrogel	MG	No	<1 mm	19.5	Normal ductal epithelium
27-3 PTCT	CD140-tdTomato+					7	5×10^5^	Hydrogel	SC (Rear)	No	N/a	19.5	N/a
28-3 PTCT	CD140-	1	Elongated, some foci	4	30d	10	5×10^5^	Hydrogel	MG	No	15.3 ± 1.5	18.5	Fibrosis, vimentin positive
28-3 PTCT	CD140-					10	5×10^5^	Hydrogel	SC (Rear)	No	12.3 ± 1.1	18.5	
27-1 PTCT	CD140-tdTomato+	1	Cobblestone, no foci	4	30d	11	5×10^5^	Hydrogel	MG	No	N/a	36.4	N/a
27-1 PTCT	CD140-tdTomato+					11	5×10^5^	Hydrogel	SC (Rear)	No	N/a	36.4	
28-6 PTCT	CD140-tdTomato+	1	Foci, rapid proliferation	4	30d	11	5×10^5^	Hydrogel	MG	No	<1 mm	42	Fibrosis, squamous epithelium
28-6 PTCT	CD140-tdTomato+					11	5×10^5^	Hydrogel	SC (Rear)	No	N/a	42	N/a
*ss*020513_1 PTCT	CD140- CD49f+	1	Cobblestone, no foci	4	102d	3	4×10^5^	Hydrogel	SC (Shoulder)	No	5.3 ± 0.6	32	Glandular, squamous epithelium, CK8/18 positive
*ss*020513_1 PTCT	CD140- CD49f+				242d	3	1×10^6^	Matrigel	SC (Flank)	Yes	6.0 ± 0.4	10	
*ss*020513_2 PTCT	CD140- CD49f+	1	Cobblestone, no foci	4		3	8×10^5^	Matrigel	SC (Flank)	Yes	6.2 ± 1.2	10	
*ss*082112_PTCT	CD140-	6	Cobblestone, no foci	4	59d	10	1×10^6^	Matrigel	MG	Yes	<1 mm	10.6	Normal ductal epithelium
*ss*082112_PTCT	CD140-	6				11	1×10^6^	Matrigel	SC (Rear)	Yes	7.21 ± 0.2	10.6	Glandular, squamous epithelium

NSG mice (*n* = 3-7), at various ages, were injected with cells at various passages post-transduction (P_I_) in hydrogel or Matrigel into mammary gland fat pads (MG) or subcutaneously (SC), with or without implanted estrogen (E) and progesterone (P) pellets. Cells were grown in the mice for up to 42 weeks and the widest diameter (±SEM) and features of growths are indicated

Pigs in study two received daily 17β-estradiol injections (IM, 0.1 mg/kg; Sigma Aldrich, St. Louis, MO) for 7d after lentivirus instillation to stimulate MEC proliferation [9]. Similarly, pigs in studies three and four received daily 17β-estradiol for 7d prior to excision of mammary tissue. Upon reinstillation of lentivirus-transduced cells, a 17β-estradiol/cholesterol pellet (0.05 mg/kg) was placed subcutaneously, reducing the need for daily hormone injections. All pigs serving as donors for mammary tissue received penicillin intramuscularly 24 h prior to tissue collection to reduce the potential bacterial contamination of cultures. NSG mice carrying pMEC lines ss020513_1 and ss020513_2 (*n* = 4) and ss082112 (*n* = 4) received a pellet containing 2 μg 17β-estradiol and 0.75 mg progesterone (Sigma-Aldrich) at the time of cell injection to promote the proliferation of engrafted cells.

### Primary mammary cell isolation

In studies one and four, immediately following exsanguination of pigs, the skin was disinfected, the nipple retracted and ~1 g of mammary tissue excised (*n* = 5 glands). In study three, pMEC were obtained by removing endogenous parenchyma from six mammary glands from five-week old pigs under isoflurane anesthesia using a cleared mammary gland procedure essentially as described [20]. Analgesic (banamine, 2–5 mg/kg) was administered postoperatively.

#### Organoid preparation

Minced mammary tissue was digested (1.5 mg/ml collagenase A (Roche, San Francisco, CA; 75 μg/ml DNase I, Roche; 1 mg/ml hyaluronidase, MP Biomedicals, Santa Ana, CA) in growth media (10 % fetal bovine serum [FBS], DMEM/F-12, penicillin G/streptomycin sulfate/amphotericin B) at 37 °C for 3 h. Organoids (40–100 μm diameter) were plated in primary porcine mammary epithelial media (modified from MEGM [21] as a 1:1 mix of MCDB170 (US Biological, Salem, MA) and DMEM/F-12 (CellGro, Manassas, VA) with penicillin G/streptomycin sulfate/amphotericin B, 0.5 % FBS, bovine insulin (7.5 μg/mL, Sigma-Aldrich), human EGF (5 ng/mL, Millipore, Billerica, MA), hydrocortisone (0.25 μg/mL, Sigma-Aldrich), human apo-transferrin (2.5 μg/mL, Sigma-Aldrich), ethanolamine (0.1 mM, Sigma-Aldrich), o-phosphoethanolamine (0.1 mM Sigma-Aldrich), bovine pituitary extract (35 μg/mL, Gemini Bio-Products, West Sacramento, CA), and lipid-rich bovine serum albumin (0.1 %, Gemini Bio-Products).

### Cell culture

Primary pMEC were maintained in porcine mammary epithelial media or growth media, and were differentially trypsinized to reduce the number of contaminating fibroblasts [21]. HEK293FT cells (Invitrogen, Grand Island, NY) were maintained in HEK293FT media (DMEM, 10 % FBS, penicillin G/streptomycin sulfate, non-essential amino acids and 1 mM sodium pyruvate). NIH/3 T3 cells (ATCC) were maintained in high glucose DMEM (Hyclone Laboratories, GE Healthcare Life Sciences, Logan UT) with 10 % FBS, 10 mM Hepes, 1 mM sodium pyruvate, penicillin G/streptomycin sulfate.

### Vectors and virus

The human elongation factor 1α (EF1α)-tdTomato and phosphoglycerate kinase (PGK)-tdTomato plasmids were generated from pLVX-IRES-tdTomato (cytomegalovirus (CMV)-tdTomato; Clontech, Mountain View, CA) by cloning the EF1α promoter from pEF6/myc-His C (Invitrogen) or the human PGK promoter (GenBank:NG_008862.1) from the pMNDU3-PGK-Luc plasmid (UC Davis Vector Core, Sacramento, CA) by PCR (Additional file 1: Figure S1A). The PGK-T antigens (Tag)-CMV-tdTomato construct (Additional file 1: Figure S1B) was generated by first constructing pLVX-PGK-Tag. IRES-tdTomato was excised from pLVX-PGK-tdTomato to give pLVX-PGK. The Tag sequences encoding mouse polyomavirus small Tag (ST), middle Tag (MT) and large Tag (LT; GenBank:J02288) were amplified from p53.A6.6 (pPY-1; ATCC, Manassas, VA) and ligated into pLVX-PGK to generate pLVX-PGK-Tag. The CMV promoter was excised from pLVX-IRES-tdTomato and ligated into pLVX-PGK-Tag to generate pLVX-PGK-Tag-CMV. The tdTomato coding sequence was amplified from pLVX-IRES-tdTomato and ligated into pLVX-PGK-Tag-CMV to generate pLVX-PGK-Tag-CMV-tdTomato. All constructs were sequence verified.

Lentiviral supernatants for PGK-Tag-CMV-tdTomato were prepared by the University of California San Francisco viral core and titrated by flow cytometry (9.3 × 10^7^ transduction units [TU]/ml). CMV-tdTomato viral supernatant was produced by triple transfection of HEK293FT cells in Opti-MEM I (Invitrogen) with packaging vector (16 μg/150 cm^2^ cells; pCMV-dR8.91, UC Davis Vector Core), envelope vector (3.2 μg/150 cm^2^ cells; pMDG-VSVG, UC Davis Vector Core) and transfer vector (16 μg/150 cm^2^ cells; pLVX-IRES-tdTomato) with Lipofectamine 2000 (Invitrogen). Lentiviral particles were concentrated by 100 kDa cut-off centrifugation (Millipore).

Viral stocks were titrated in HEK293FT cells using qPCR [22]. The woodchuck hepatitis virus posttranscriptional regulatory element (WPRE) was used to measure integration and values normalized to the number of human albumin copies (WPRE Fwd GCGTCTGGAACAATCAACCT and Rev GGCATTAAAGCAGCGTATCC; hAlbumin [GenBank:152112963] Fwd GTGCTGCCTCGTAGAGTTTTCTG and Rev TCAATAGCCATGTGACCAGTGACT).

### Fluorescence activated cell sorting

Primary pMEC cultures were expanded for 13-14d post-isolation with two differential trypsinizations using Accutase (Innovative Cell Technologies, Mira Mesa, CA) to remove fibroblasts, followed by Accumax (Innovative Cell Technologies) to dislodge pMEC. Single cells were incubated with phycoerythrin-conjugated anti- CD140a (BD Biosciences, Franklin Lakes, NJ) and/or biotinylated anti-human CD49f (AbD Serotec, Oxford, UK), followed by streptavidin-Alexa 488 (Jackson Immunoresearch) and propidium iodide (10 μg/ml) and sorted using a MoFlo cell sorter (Cytomation, West Lafayette, IN).

### Viral transduction *in vitro*

Adherent pMEC were transduced overnight using a multiplicity of infection (MOI) of 100 for PGK-tdTomato, EF1α-tdTomato or CMV-tdTomato, or an MOI of 20 for PGK-Tag-CMV-tdTomato, along with polybrene (6 μg/mL; Millipore). For study four, cultures of CD140a- pMEC were transduced with PGK-Tag (ss071712), pLVX-PGK-Tag-CMV-tdTomato (ss082112, 27–1, 27–3, 28–3) or CMV-tdTomato (ss071712). Two pMEC lines (ss020513_1, ss020513_2) were sorted to be CD140a-/CD49f + then transduced with PGK-Tag-CMV-tdTomato. A subset of CD140a- pMEC transduced with PGK-Tag-CMV-tdTomato were sorted for tdTomato + either 7d (27–3, 28–3) or 17d later (27–1, 28–6) depending on the initial number of CD140a- pMEC.

### Viral transduction *in vivo*

In study two, saline or lentivirus (CMV-tdTomato, 5 × 10^6^ TU; EF1α-tdTomato, 1 × 10^7^ TU and PGK-tdTomato, 5 × 10^6^ TU with or without 6 μg/ml polybrene was instilled into the left (with polybrene; one gland/treatment) and right (without polybrene; one gland/treatment) thoracic and abdominal mammary glands of pigs (*n* = 9) via one of the two mammary ducts (intraductal) under isoflurane anesthesia. Additionally, saline or lentivirus suspension with 6 μg/ml polybrene was injected directly into the mammary parenchyma (20–25 mm subcutaneously; intramammary) in the four remaining mammary glands of each pig (one gland/treatment). Mammary glands were harvested at necropsy 5, 10 or 15 d later, and snap frozen or fixed in 4 % paraformaldehyde.

### *Ex vivo* transduction and grafting

In study three, dissociated mammary organoids (*n* = 30) were transduced overnight (MOI = 100) with PGK-tdTomato, CMV-tdTomato, EF1α-tdTomato, CMV-Tag, EF1α-Tag, PGK-Tag or no vector (control) with polybrene (6 μg/ml). After 24 h (*n* = 4 pigs) or 8d (n = 6 pigs) cultures of organoids were trypsinized and resuspended in serum-free media. Donor pigs were anesthetized with isoflurane and cells reinstilled via two intramammary injections/gland (0.9–4.5×10^5^ cells/injection; *n* = 2 glands/construct/pig) and each site closed with Vetbond (3 M, St. Paul, MN). The isografted mammary glands were harvested 3–5 weeks later, minced, and randomly divided for snap freezing in liquid N_2_ or fixation in 4 % paraformaldehyde.

In study four, lentivirus-transduced pMEC were injected into NSG mice under isoflurane anesthesia. A range of 0.4-1 × 10^6^ pMEC resuspended in 20 μL of HyStem-C hydrogel (*n* = 46 injected sites; Glycosan Biosystems, Alameda, CA) or Matrigel HC (*n* = 24 injected sites; BD Biosciences) was injected either subcutaneously or directly into the mammary gland. For instillation of cells into the mammary gland, a small skin incision (~5 mm) was made to visualize accurate placement within the mammary fat pad. Mice were treated postoperatively with a single dose of analgesic (buprenorphine; 0.05 mg/kg) and were monitored once daily over 7d for changes in health and behavior. Table 1 summarizes the mice and cell injections used.

### *In vitro* assays

Cell number was assayed using a methylene blue assay [23]. Cells were plated in 96 well-plates at 2000 cells/well (*n* = 6/cell line) on d0, and medium changed every 2d.

Cells (20,000/well) were resuspended in 0.35 % agar in growth medium and poured onto a base layer (0.7 % agar in growth media), with growth medium changed every 2d. After 21d, cells were stained (0.04 % crystal violet, 2.1 % citric acid), imaged and colonies >50 μm counted using ImageJ (NIH; http://rsb.info.nih.gov/ij/).

### Mammary gland and tumor whole mounts

Semi-thick tissue sections were dehydrated through graded ethanols to xylene (study three) or graded glycerol (study four) as described [24]. Sections were imaged using a fluorescent dissecting microscope. The percent red area was calculated using Image J. Regions positive for red fluorescence were dissected and processed to paraffin for hematoxylin and eosin staining (H&E) and immunohistochemistry. Regions having dense ductal structures (study three, PGK-Tag mammary glands) were microdissected, paraffin-embedded and sectioned for histology (H&E) and genomic DNA extraction.

### Western blotting

Cells (1–3 × 10^6^) were lysed and sonicated in buffer with protease and phosphatase inhibitors, and western blots performed as described [25]. Antibodies were from Santa Cruz Biotechnology (rat polyomavirus early, cyclin D1, and p53), Cell Signaling Technology (Rb, phospho Rb, MAPK1/3 and phospho MAPK1/3)) and Jackson ImmunoResearch (HRP-conjugated secondary antibodies).

### Genomic DNA extraction and PCR detection of integration

Genomic DNA was extracted from paraffin-embedded tissues as described [26] using combined heating and non-heating protocols. DNA quality was assessed using primers for the porcine prolactin receptor gene [27]. The incidence of lentiviral integration in studies two and three was determined using primer sets specific to tdTomato (Clontech; tdTomatoFin Fwd CTCCGAGGACAACAACATGG and Rev CTTGGTCACCTTCAGCTTGG; CMVtdTomato Fwd AACACGATGATAATATGGTGAGCAAGGG and TdTomatointernal Rev GACAGCTTCTTGTAATCGGGGATGTC) or amplified a product specific for PGK-Tag (PGKTagspec5P TGAAGATGTAAAGGGTCAAATAGC and PGKspec3P-2 AAGGCATTAAAGCAGCGTATC).

#### RT-qPCR and qPCR (study two)

Total RNA was extracted and reverse transcribed as described [28]. Primers spanned across exons of LEF1 (Fwd GACGAGCACTTTTCTCCAGGA, Rev TAATCTGTCCAACACCACCCG [GenBank:XM_005666939]), cyclin D1, (Fwd CCCTCCGTGTCCTACTTCAA, Rev CAGGCGGCTCTTTTTCAC [GenBank:AK400348)], cyclin A2, (Fwd TTGTGGGCACTGCTGCTATG, Rev GCAAGGACTTTCAAAACGAGGTG [GenBank:GQ265874]), MYC, (Fwd CGCTTTTTGGACGCTGGATT, Rev TTCTCCTCCTCGTCGCAGTA [GenBank:X97040]), RB1 (Rb), (Fwd ACGCCAACAAAAATGACTCC, Rev GTTGCCTCCTTCAGCACTTC [GenBank: JX099502]), TP53 (p53), (Fwd CCATCCTCACCATCATCACACT, Rev CTCTGTGCGGCGGTCTCT [GenBank:NM_213824]), P21, (Fwd GCAGACCAGCATGACAGATT, Rev TGTTTCCAGCAGGACAAGG [GenBank:XM_001929558]), P16, (Fwd GAGGGCTTCCTGGACACTTTG, Rev TGCAGTATCTCTGGGTTTCAATGA; [GenBank:AJ316067]) and 18S ribosomal RNA, (Fwd ACGGCTACCACATCCAAGGA, Rev CCAATTACAGGGCCTCGAAA [GenBank: AF179868]). All PCR products were sequenced. RT-qPCR was as described [28], where relative transcript abundance was calculated using a 5-point standard curve obtained by 5-fold serial dilutions of a pMEC complementary DNA pool. The average relative expression for each sample was normalized to 18S ribosomal RNA levels [29].

DNA was purified from tissues homogenized in Tri-Reagent (Molecular Research Center, Inc, Cincinnati, Ohio). Genomic DNA (40 ng) was amplified with tdTomatoFin primers using qPCR [28]. Standard curves were generated from genomic DNA extracted from mouse mammary tumor cells (SSM-2) transduced with EF1α-tdTomato lentivirus and selected for tdTomato expression using a MoFlo cell sorter. The relative number of integrated virus particles was normalized to the corresponding level of 18S ribosomal DNA as a loading control for gene copy number (Fwd ACGGCTACCACATCCAAGGA, Rev CCAATTACAGGGCCTCGAAA [Genbank: NR_046261]).

### Immunohistochemistry

Slides were prepared as described [28], with modifications. Sections were incubated with anti-dsRED (1:50; Clontech), anti-human progesterone receptor (1:50; DakoCytomation, Carpinteria, CA), anti-human estrogen receptor (clone 6 F11, ThermoFisher Scientific, Waltham, MA), anti-vimentin (1:100; Millipore), anti-bovine cytokeratin 8/18 (1:2000; Fitzgerald Industries, Acton, MA), or anti-Ki67 (clone Ab-4; 1:1000; ThermoFisher Scientific) in 5 % horse serum in PBS at 4 °C overnight and detected with NovaRED (Vector Laboratories) or DAB (Invitrogen).

### Statistics

Differences were assessed by two-way ANOVA, and *P*-values calculated using Students *t*-tests. Data is presented as means ± SEM, with significance at *P* < 0.05. For animal studies, individual mammary glands were treated as the experimental unit. Animal group sizes were selected to provide >80 % power to detect differences, taking into consideration our experience with assessing porcine mammary gland morphology [29] and the typical engraftment characteristics and rates of primary bovine and human MEC in immunodeficient mice [30, 31].

## Results

### Primary culture of porcine mammary cells

**Fig. 1:**
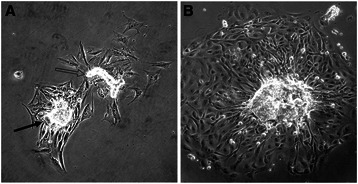
Representative images of pig mammary organoids collected after enzymatic dissociation of mammary gland tissue from a non-pregnant female. **a** A cluster of epithelial cells (closed arrowhead) and piece of duct (open arrowhead) with surrounding outgrowth 24 h after dissociation and plating. **b** An organoid with typical outgrowth 48 h after dissociation. Scale bar = 100 μm

**Fig. 2:**
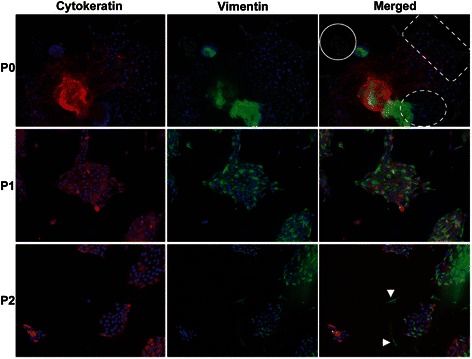
Representative fluorescence images from pig mammary organoids 48 h after dissociation (P0) and after passages 1 and 2 (P1 and P2). Four distinct populations of cells were visible at P0. Cytokeratin-positive luminal epithelial cells, vimentin-positive fibroblasts (dashed circle, arrowheads in P2), cells positive for both vimentin and cytokeratin (dashed rectangle) and small, vimentin- positive cells (solid circle) found infrequently only at P0

Dissociation of mammary tissue from nulliparous pigs (study one) yielded epithelial organoids that adhered to plastic within 24 h (Fig. 1). At 2d post-dissociation, mixed populations of cells included a cytokeratin-positive epithelial population (luminal), cells positive for both cytokeratin and vimentin (basal/myoepithelial) and two morphologically distinct vimentin-positive populations, one most likely being fibroblasts (Fig. 2).

### Lentivirus for manipulating pMEC *in vitro* and *in vivo*

**Fig. 3:**
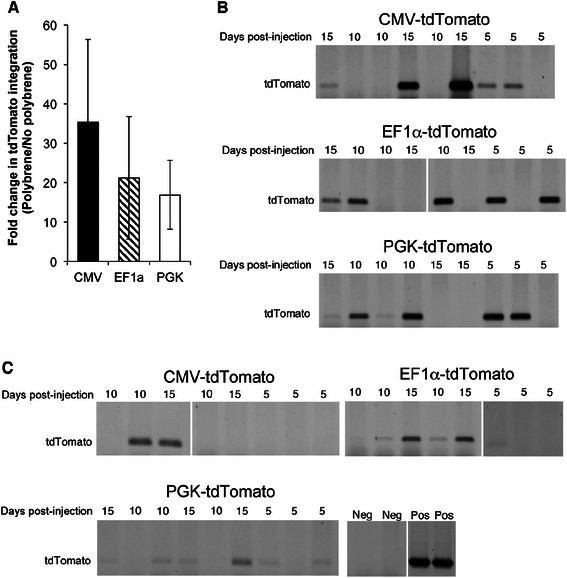
Injection of lentivirus into the pig mammary gland. **a** Glands were injected intraductally (*n* = 9 pigs) with CMV-tdTomato, EF1α-tdTomato or PGK-tdTomato lentivirus with or without polybrene and harvested 5, 10 or 15d later. Lentiviral integration was determined by qPCR for tdTomato, corrected for 18S ribosomal RNA levels and expressed as a ratio of tdTomato integration with or without polybrene. Data are means ± SEM (*n* = 6-7). **b** Glands were injected intraductally (*n* = 9 pigs) with CMV-tdTomato, EF1α-tdTomato or PGK-tdTomato lentivirus and polybrene and harvested 5, 10 or 15d later. **c** Injections were into the mammary parenchyma (*n* = 9 pigs) with CMV-tdTomato, EF1α-tdTomato or PGK-tdTomato lentivirus and polybrene and harvested 5, 10 or 15d later. Negative controls (Neg) are genomic DNA from the mammary glands of two untreated pigs. Positive controls (Pos) are two pMEC lines transduced with CMV-tdTomato

We compared the CMV, EF1α and PGK promoters in lentivirus-transduced pMEC (study one), and determined EF1α to be the most effective *in vitro* (Additional file 2: Figure S2B). We next determined which promoter was most effective for pMEC *in vivo*, and the best route (intraductal or intramammary) for introducing lentivirus into the mammary gland (study two). Analysis of genomic DNA revealed that polybrene increased the incorporation of lentivirus instilled intraductally by 24-fold (Fig. 3a; *P* < 0.05). We detected tdTomato in 5/9 CMV-, 5/9 EF1α- and in 4/9 PGK-tdTomato glands injected intraductally with lentivirus (Fig. 3b). In mammary glands receiving intramammary injections of lentivirus with polybrene we detected tdTomato in 2/8 CMV-, in 4/8 EF1α- and in 5/9 PGK-tdTomato glands (Fig. 3c). Glands transduced by intraductal instillation were analyzed for ductal outgrowths expressing tdTomato (Additional file 3: Figure S3). Clustered tdTomato-positive structures were present in the mammary gland injected with either EF1α-tdTomato or CMV-tdTomato lentivirus, consistent with localized transduction.

**Fig. 4:**
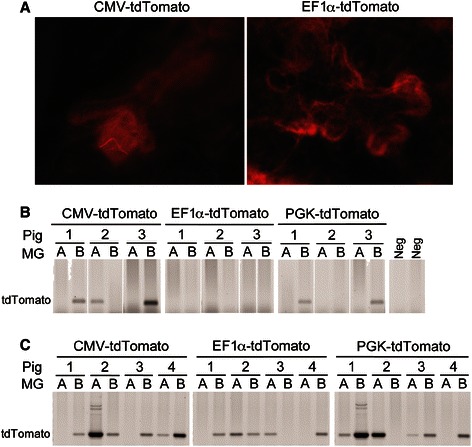
Installation of lentivirus-transduced pig mammary epithelial cells (pMEC). **a** Representative images of red fluorescent terminal ductal lobular units identified in whole mounts of mammary glands injected with pMEC 8d after the cells were transduced with CMV-tdTomato, EF1α-tdTomato or PGK-tdTomato lentivirus. Tissues were harvested 3 or 5 weeks later. **b** Detection of tdTomato by PCR of genomic DNA from mammary glands analyzed in (**a**). Negative controls (Neg) are genomic DNA from the mammary glands of two untreated pigs. **c** Detection of tdTomato by PCR of genomic DNA from mammary glands injected with pMEC 24 h after the cells were transduced with CMV-tdTomato, EF1α-tdTomato or PGK-tdTomato lentivirus. Tissues were harvested 4 weeks later

We examined whether pMEC transduced *ex vivo* would develop into epithelial structures upon transplantation into donor pigs (study three). Expansion of cells for 8d post-transduction before reinstallation yielded fluorescent TDLU outgrowths from EF1α-tdTomato (*n* = 1 of 8 mammary glands) and CMV-tdTomato (*n* = 2 of 8) transduced cells (Fig. 4a), but not from PGK-tdTomato transductants or in control glands (not shown), despite detection of tdTomato in 2/6 glands transplanted with PGK-tdTomato pMEC (Fig. 4b). Expansion of cells for 24 h post-transduction prior to reinstallation led to detection of genomic tdTomato in >50 % of glands injected with CMV-tdTomato pMEC, EF1α-tdTomato or PGK-tdTomato transduced cells (Fig. 4c).

### Oncogene-induced pMEC transformation *in vitro*

**Fig. 5:**
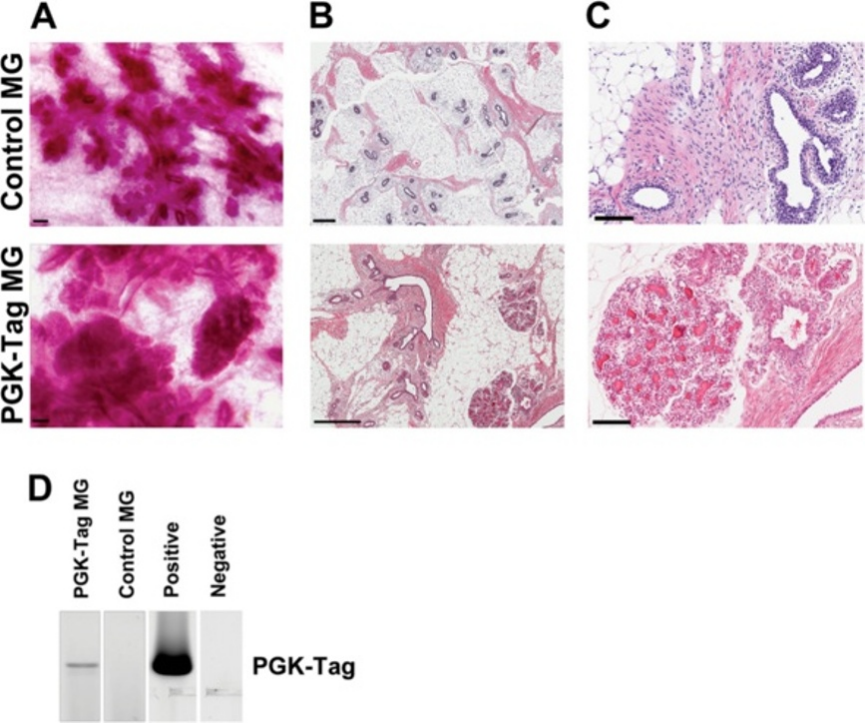
PGK-Tag transformed pMEC promote the precocious development of dense epithelial structures that resemble terminal ductal lobular units (TDLU). **a** Whole mounts of mammary glands injected with either pMEC transduced with PGK-Tag lentivirus or non-transduced pMEC (control mammary gland). **b** Densely packed structures identified by whole mount analysis of PGK-Tag engrafted mammary glands were sectioned and stained (H&E). A parenchyma-rich area from a contralateral control mammary gland is included for comparison. Scale bar =100 μm. **c** Magnification of H&E from (**a**) scale bar = 100 μm. **d** PCR detection of PGK-Tag in genomic DNA from paraffin sections of the mammary gland shown in (**b**). Positive control is genomic DNA from pMEC transduced with PGK-Tag. Negative is ddH_2_O template

We compared the efficacy of the three promoters for expressing the Tag oncoproteins ST, MT and LT produced by splicing of the murine polyomavirus Tag. Based on the number of colonies in soft agar, the PGK promoter was most effective for directing Tag-induced transformation of pMEC *in vitro* (Additional file 4: Figure S4). When pMEC transduced with PGK-Tag, CMV-Tag or EF1α-Tag were injected as isografts, we only detected dense structures in whole mounts from all PGK-Tag engrafted glands that were evaluated (Fig. 5a; 2 pigs, 4 mammary glands total). These structures histologically resembled TDLU (Figs. 5b and c), and were positive for the expression of estrogen receptor (Additional file 5: Figure S5A), progesterone receptor (Additional file 5: Figure S5B), and epithelial cytokeratins (Additional file 5: Figure S5D) and negative for vimentin (Additional file 5: Figure S5C). Areas within and surrounding the TDLU were confirmed to be PGK-Tag positive (Fig. 5d). Subsequent experiments involving Tag utilized the PGK promoter.

**Fig. 6:**
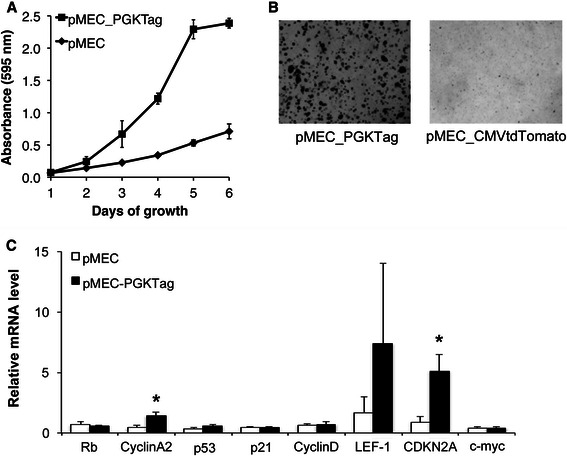
Pig mammary epithelial cells (pMEC) transduced with PGK-Tag lentivirus exhibit a transformed phenotype. **a** Growth of pMEC transduced with PGK-Tag (*n* = 4) and control pMEC transduced with CMV-tdTomato (*n* = 3). **b** Representative image of PGK-Tag transduced pMEC in soft agar. PGK-Tag pMEC formed 753.3 ± 30 colonies/well while CMV-tdTomato pMEC yielded no colonies. **c** pMEC transduced with PGK-Tag (*n* = 4) or PGK-Tag-CMV-tdTomato (*n* = 1) had similar expression of cyclin D1, myc, p53 and Rb and increased expression of cyclin A2 and p16 (*P < 0.05) compared to control pMEC

**Fig. 7:**
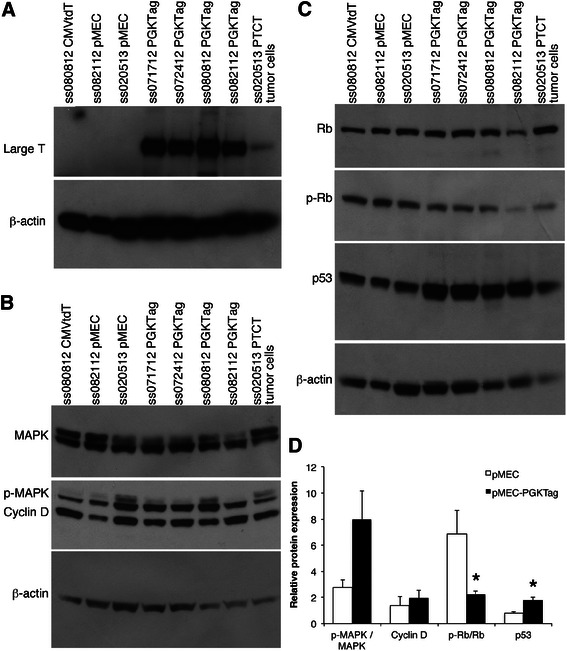
PGK-Tag transduced pMEC have altered oncogene expression. **a** Proteins from pMEC (*n* = 3), pMEC-PGKTag (*n* = 4) and an NSG mouse tumor from injection of pMEC-PGK-Tag-CMV-tdTomato cells (*n* = 1) were analyzed by western blot for ST, MT and LT. **b** Western blot of the same proteins using **b**) anti-MAPK1/3, anti-phospho MAPK1/3, anti-cyclin D1 and anti-β-actin antibodies, and **c**) anti-Rb, anti-phospho Rb, anti-p53 and anti-β-actin. **d** Protein levels from blots in **b**) and **c**) were quantified for pMEC (*n* = 3) and pMEC-PGKTag cells (*n* = 5)

We next profiled Tag-induced molecular changes in pMEC. Those pMEC (*n* = 4 pigs) transduced with PGK-Tag exhibited increased proliferation and anchorage-independent growth (Figs. 6a-b). Analysis of the expression of oncogenes (LEF-1, cyclin A2, cyclin D1, myc) and tumor suppressor genes (p16, p21, Rb and p53) revealed that Tag-transduced pMEC had elevated P16 (*P* = 0.01) and cyclin A2 mRNA expression (*P* = 0.03; Fig. 6c). The LT protein was detected in PGK-Tag transduced pMEC (Fig. 7a), with upregulated TP53 (*P* = 0.007) and decreased phosphorylated Rb (*P* = 0.01; Figs. 7c-d) and a tendency for increased phosphorylated MAPK1/3 (*P* = 0.13; Fig. 7b). We also refined our transduction protocol using a vector that co-expressed tdTomato with Tag (PGK-Tag-CMV-tdTomato). We found that 55 ± 7 % pMEC transduced by PGK-Tag-CMV-tdTomato were red 7d after transduction whereas 95 ± 0.5 % were positive for tdTomato 4 weeks post-transduction (not shown).

### FACS sorting of primary pMEC

We separated pMEC using lineage-specific markers previously used for human and mouse MEC [32]. Stromal cells were removed by sorting for CD140a. The remaining pMEC were sorted as CD49f + and CD49f- that comprised 79 % and 21 %, respectively (*P* < 0.001). The CD140a-CD49f- cells were enriched for cytokeratin-positive and vimentin-negative cells (luminal-like) whereas CD140a-CD49f + subpopulations were enriched for cytokeratin- and vimentin-positive cells (basal-like) (Additional file 6: Figure S6). Few cytokeratin-negative and vimentin-positive cells were present in CD140a^-^ CD49f + populations (0.07 % +/- 0.03, second passage).

Dissociated pMEC depleted for CD140a (CD140a-, *n* = 5; Table 1) and enriched for CD49f (CD140a-CD49f + *n* = 2; Table 1) were transduced with PGK-Tag-CMV-tdTomato, and some further enriched for tdTomato (CD140a- tdTomato+; *n* = 3; Table 1). Cells sorted for CD140a- tdTomato + exhibited red fluorescence *in vitro* (Additional file 7: Figure S7A). Transduction by PGK-Tag-CMV-tdTomato yielded transformed pMEC that gave rise to colonies able to grow in soft agar (Figure S7B) expressing ST, MT and LT (not shown).

Populations of pMEC transduced by PGK-Tag-CMV-tdTomato varied morphologically *in vitro*. While CD140a-CD49f + pMEC retained a cobblestone morphology (Additional file 8: Figure S8A), CD140a^-^tdTomato^+^ pMEC were elongated (Additional file 8: Figure S8B), developed foci (Additional file 8: Figure S8C) or maintained a cobblestone morphology without foci (Additional file 8: Figure S8D).

### Transformed xenografted pMEC generate orthotopic and ectopic tumors

To determine the tumorigenicity of transformed pMEC, all cell lines were injected into NSG mice either subcutaenously with hydrogel or Matrigel, or into the mammary fat pads. Cells injected in the fat pad, either in Matrigel or hydrogel, failed to form tumors after 36 weeks (*n* = 5; Table 1). There were striking differences among tumors that formed subcutaneously following co-injection with Matrigel or hydrogel. While all transformed pMEC in Matrigel developed tumors (>1 mm) after 4 weeks, only one line in hydrogel developed tumors after 16 weeks (Table 1).

**Fig. 8:**
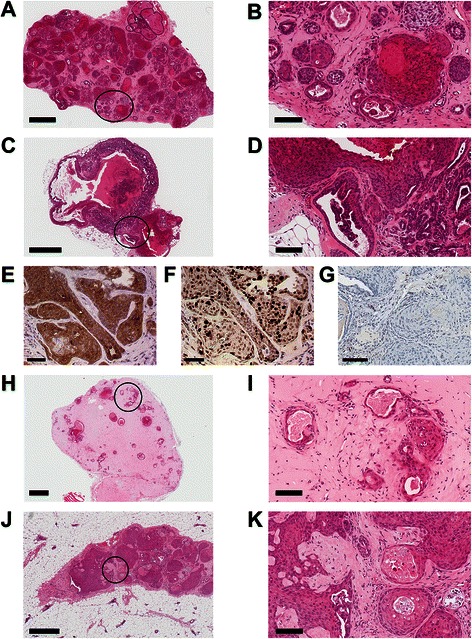
Xenografted CD140-CD49+ pMEC transduced with PGK-Tag-CMV-tdTomato injected subcutaneously with Matrigel or hydrogel produce epithelial neoplasms. **a** Section (H&E) from a CD140-CD49+ PGK-Tag-CMV-tdTomato tumor in Matrigel showing neoplastic glandular epithelium. Scale bar = 600 μm. **b** Magnified encircled area in (**a**). Scale bar = 100 μm. **c** Section (H&E) from a CD140-CD49+ PGK-Tag-CMV-tdTomato tumor in hydrogel showing neoplastic glandular epithelium. Scale bar = 600 μm. **d** Magnified encircled area in (**c**). Scale bar = 100 μm. Immunolocalization of (**e**) cytokeratins 8/18, (**f**) Ki67 and (**g**) estrogen receptor in CD140-CD49+ PGK-Tag-CMV-tdTomato tumorous pMEC injected with Matrigel. Scale bar = 100 μm. **h** CD140- pMEC transduced with PGK-Tag-CMV-tdTomato develop ductal-like structures as subcutaneous xenografts. Section (H&E) depicting scattered glandular epithelium with hollow or cell-filled lumens. Scale bar = 600 μm. **i** Magnified encircled area in (**h**). Scale bar = 100 μm. **j** CD140-tdTomato + pMEC transduced with PGK-Tag-CMV-tdTomato produce epithelial-type neoplasms in mammary fat pads. Section (H&E) depicting fibrosis with neoplastic squamous epithelium. Scale bar = 600 μm. **k** Magnified encircled area in (**j**). Scale bar = 100 μm

Tumors from CD140a^-^CD49f + PGK-Tag-CMV-tdTomato pMEC injected with Matrigel or hydrogel were 54–74 % positive for red fluorescence (Additional file 9: Figure S9A-B). Tumors comprised mixed neoplastic glandular epithelium and nests of squamous epithelium having intracellular bridges and dyskeratosis with occasional microcalcifications and dense fibrosis (Fig. 8a-d). Immunohistochemistry for cytokeratin 8/18, Ki-67 and nuclear hormone receptors confirmed these tumors were epithelial and proliferative (Figs. 8e-f), albeit negative for estrogen receptor (Fig. 8g) and progesterone receptor (not shown). Tumors arising from CD140a- PGK-Tag-CMV-tdTomato pMEC co-injected subcutaneously with Matrigel were <50 % red (Additional file 9: Figure S9E) and contained occasional nests of squamous epithelium mixed with glandular epithelium (Fig. 8h-i). Whole mount analysis revealed red fluorescent growths in the mammary fat pads of mice carrying CD140a- pMEC in Matrigel (6/8 injections; Additional file 9: Figure S9D) that were subsequently found to comprise non-neoplastic ducts or cysts (Fig. 8f), similar to results for normal human and bovine MEC transplanted into the mouse mammary fat pad [31, 30].

We recorded variation among growths arising from CD140^-^ pMEC instilled with hydrogel. One line of transformed pMEC only yielded microscopic fluorescent growths (Additional file 9: Figure S9E), which contained fibrosis and neoplastic squamous epithelial cells (Fig. 8j-k). A second line developed palpable mammary and subcutaneous tumors that were comprised chiefly of fibrous connective tissue. Accordingly, these tumors were strongly vimentin-positive with only scant dsRED immunoreactivity (not shown). A third line of CD140a-tdTomato + pMEC injected in hydrogel infrequently developed tumors in the mammary fat pad (1/8 injections; not shown) that comprised non-neoplastic ducts (not shown) similar to those found for pMEC co-instilled with Matrigel (Fig. 8h).

## Discussion

Here we report the first successful lentivirus-mediated transgenesis and transformation of primary pMEC *in vitro* and *in vivo*. Given significant structural and functional similarities between the mammary glands of pigs and humans [9], our approach is a first step toward a promising animal model in which to investigate the tissue-level cellular and environmental interactions behind human breast development and oncogenesis.

A variety of genetically-engineered mouse models has enabled the identification of various genes involved in mammary cancer initiation and progression, yet the resulting tumors often differ in their pathology compared to human breast cancers [33]. This discordance may reflect the greater frequency at which mouse tumors develop from alveolar structures [33] compared to those of the human breast that often arise from less-differentiated TDLU-1 and -2 [17]. As such, investigations into the initiation and progression of tumors within TDLU have been hampered by the absence of similar structures in the mouse mammary gland alongside the challenges associated with obtaining human tissues. Development of a pig breast cancer preclinical model stands to complement recent advances to optimize the limitations of mouse models such as the addition of human stromal elements to the mouse mammary fat pad [34] and reconstitution of the mouse mammary epithelium with human preneoplastic cells [35].

Our techniques enable transformation of MEC derived from the TDLU of pre- and peripubescent pigs that are synonymous with TDLU-1 and -2 in the human breast [9]. Moreover, vacuum-assisted mammary biopsies [19], which are impractical in rodents [36], will allow serial sampling of a pig model of breast cancer to study the response to cancer treatment over time, or the influence of environmental and lifestyle factors on breast cancer risk. While a porcine pre-clinical model has limitations including increased cost and housing requirements [36], these drawbacks are countered by the potential ability to authentically model normal human breast development and breast cancers within a context that is biologically and physiologically more similar to the human.

The human breast and pig mammary gland have a similarly complex stromal microenvironment, which contrasts to that of the rodent mammary gland [18]. Our results highlight the influence of stromal factors on the occurrence and rate of pMEC tumor formation *in vivo*. Similar to transformed human breast epithelial cells [37], transformed pMEC failed to form tumors in the microenvironment of the mouse mammary fat pad despite developing tumors ectopically. Our data corroborates previous evidence showing that elements of the mouse mammary fat pad are inadequate to support proliferation of non-native epithelium [18]. These data, alongside work by others [34, 31, 30], adds to evidence highlighting differences between species in the stroma-directed behavior of the mammary epithelium. Indeed, attempts to reconstruct human [31] or bovine [30] mammary tissues in the mouse mammary fat pad by xenografting tissue fragments or dissociated MEC failed to generate species-specific TDLU.

Given that the pig mammary gland is rich in connective tissues, co-inoculating irradiated pig stromal fibroblasts with transformed pMEC may have increased their viability when xenografted into the mouse mammary fat pad, as for human and bovine MEC [34, 38]. Along these same lines, Matrigel promoted transformed pMEC to form tumors, consistent with the stromal requirements of xenografts of human breast cancer cells [39]. The matrix proteins found in Matrigel can support the growth of cells in a foreign environment, recapitulating the connective-tissue rich stroma of the human breast and pig mammary gland [18, 40]. However, unlike in humans, mammary carcinomas are extremely rare in pigs [41]. This low incidence may reflect innate repressive effects of the stroma, which can restrain the tumorigenicity of undifferentiated mouse embryonic carcinoma cells [42] and prevents marginally abnormal human mammary organoids developing hyperplasias in humanized mouse mammary fat pads [34]. The structure and morphology of tumor stroma associated with xenografts differs from normal stroma [43], suggesting that changes within tumor stroma may be more permissive. Accordingly, transformed porcine dermal fibroblasts readily formed undifferentiated sarcomas in the mammary glands of immunosuppressed pigs [44], while we found that isogenic transplanted pMEC transformed by Tag failed to form palpable tumors in immunocompetent pigs (data not shown). While immunosuppression may have increased the take of transformed pMEC [44], it is also possible that transformation of stromal cells is also required to induce mammary carcinomas in pigs. As such, future work to overcome these repressive mechanisms and develop the pig as an authentic breast cancer model may open up hitherto unexplored mechanisms to defeat breast cancer.

We transformed pMEC by lentivirus-mediated expression of murine Tag proteins. While Tag or similar viral oncoproteins have not been directly implicated in human breast carcinogenesis, Tag activates various oncogenes and bypasses p53 and Rb [45, 46] as in many human breast cancers [47, 48]. Mammary tumors that develop from germline transgenic MT expression in the mouse mimic invasive human breast carcinomas with progressive loss of ER coincident with increased invasiveness [49]. Herein, Tag-transformed pMEC developed estrogen receptor- negative, proliferative tumors when xenografted to mice. Interestingly, pMEC co-injected with Matrigel into estrogen-treated mice displayed a shorter latency to tumor formation, raising the possibility that transformed pMEC may have initially been hormone-sensitive. Given that mice carrying pMEC co-injected with hydrogel were not treated with estrogen, we cannot ascribe any differences in tumor formation rates to hormone supplementation. Nevertheless, our observations are consistent with similar findings in a mouse explant model of transgenic MT [50].

Previous reports indicate that primary human MEC require disruption of multiple pathways for complete transformation [37, 51, 35] whereas rodent cells can be transformed by two oncogenes [52]. Along these lines, primary pig fibroblasts [44] and human breast epithelial cells [51] were transformed by sequential transduction with dominant negative p53, activated CYCLIN-dependent kinase complex (Cyclin D1/CDK4), c-Myc, H-Ras and telomerase. Our approach provides for the efficient induction of tumorigenesis in primary pMEC by lentivirus-mediated expression of Tag, which upregulates multiple oncogenic pathways. This approach led to the formation of heterogeneous tumors, perhaps as a consequence of lentiviral transduction. Along these lines, transgenic expression of MT in the mouse mammary gland induced homogenous luminal, solid adenocarcinomas [53] whereas low levels of lentivirus-directed MT transformed both luminal and basal MEC, leading to concomitant heterogeneity among the tumors [54]. One further explanation may be variable Tag expression within pMEC subpopulations. Consistent with this postulate, the degree of Ras expression in transformed primary human MEC correlated with tumorigenicity *in vivo* [37]. Interestingly human MEC transformed with Ras-SV40 ST/LT-hTERT formed poorly differentiated squamous tumors when xenografted to the mammary fat pad of immunocompromised mice [37], similar to our findings with xenografted pMEC transformed by Tag. Although squamous differentiation is rare among human breast carcinomas, it is a phenotype that has been linked to Wnt activation [55], which has been associated with basal-like and receptor negative breast cancers [56]. Transformation of pMEC by alternative oncogenes may yield tumors with distinct histopathologies and hormone receptor profiles. In addition, our data indicate that selecting pMEC for CD49f + enhanced the subsequent formation of luminal breast carcinomas, yielding tumors with luminal differentiated epithelium, whereas unenriched pMEC developed as normal ductal structures or fibrous tumors with and without squamous epithelium. It is possible that selection for CD49+/EpCAM+ cells in combination with culture conditions to increase ErbB3 expression over EGFR expression may increase the likelihood of generating luminal adenocarcinomas [35].

## Conclusions

We have developed an approach for transforming porcine mammary epithelium. Our data point to similarities between the responses of pMEC and human MEC when xenografted to the mouse mammary fat pad, highlighting candidate differences in stromal requirements for oncogenesis across species. These studies lay the basis for investigating complex interactions underlying human breast cancer initiation and progression.

## Additional files

Additional file 1: Figure S1. Plasmid maps of lentiviral vectors. (A) Map of the construct pLVX-IRES-tdTomato (CMV-tdTomato) wherein the CMV promoter directs tdTomato expression. The CMV promoter was replaced by the EF1α or PGK promoter to create EF1α-tdTomato or PGK-tdTomato. (B) To generate the pLVX-PGK-Tag-CMV-tdTomato vector, the pLVX-PGK-Tag vector was first created by replacing the IRES and tdTomato from the vector PGK-tdTomato with murine polyomavirus T antigen that encodes small T (ST), middle T (MT) and large T (LT) antigens. The CMV promoter and tdTomato coding sequence were sequentially added to the vector, creating a pLVX-PGK-Tag-CMV interim vector and the final expression vector pLVX-PGK-Tag-CMV-tdTomato. (PDF 155 kb)
Additional file 2: Figure S2. Transduction of pMEC by CMV-, EF1α- and PGK-TdTomato lentivirus. (A) Representative bright field and corresponding fluorescent images of primary pMEC at passage 0, 7d after transduction by CMV-tdTomato, EF1α-tdTomato or PGK-tdTomato lentiviral constructs. (B) Quantification of cells expressing tdTomato, as a measure CMV, EF1α and PGK promoter activity in pMEC. (PDF 1353 kb)
Additional file 3: Figure S3. Lentiviral integration following intraductal injection of lentivirus encoding CMV-tdTomato, EF1α-tdTomato or PGK-tdTomato (*n* = 9 pigs/lentiviral construct). Mammary tissues were harvested 5d post-injection, processed into paraffin and sections analyzed by immunohistochemical detection of dsRED (monomer of tdTomato) with using NovaRed for detection and a hematoxylin counterstain. The control section was not exposed to the dsRED antibody. The negative section is from a gland that was not injected with tdTomato expressing lentivirus. Scale bar = 100 μm. (PDF 624 kb)
Additional file 4: Figure S4. The promoter of large, middle and small T antigen (Tag) expression affected the rate of anchorage-independent colony formation. Representative images from three replicate experiments to measure soft-agar colony formation by pMEC (*n* = 2 wells/construct) transduced with CMV-Tag, EF1α-Tag, PGK-Tag or CMV-tdTomato lentivirus (MOI of 100). NIH-3 T3 cells were transduced with the same constructs as a positive control. Cells transduced by CMV-tdTomato served as negative control. (PDF 508 kb)
Additional file 5: Figure S5. Immunohistological features of the dense epithelial structures that appeared after isotopic engraftment of PGK-Tag transformed pMEC. Representative images detailing the expression of (A) estrogen receptor (B) progesterone receptor (C) vimentin and (D) cytokeratin. Scale bar = 100 μm. (PDF 9416 kb)
Additional file 6: Figure S6. Vimentin and cytokeratin expression in CD49f-/+ populations. Representative images depicting cytokeratin (red) and vimentin (green) expression in FACS sorted pMEC. Cells affixed to glass slides were stained by immunofluorescence for both pan-cytokeratin and vimentin. (A) CD140-CD49- pMEC. (B) CD140a-CD49f + pMEC at passages 2 (P2), 3 (P3) and 5 (P5). Vimentin-only positive cells are circled. (PDF 846 kb)
Additional file 7: Figure S7. Porcine mammary epithelial cells (pMEC) were transduced with PGK-Tag-CMV-TdTomato (PGK-Tag-CMV-tdT) lentivirus, cultured for 7d then sorted using a MoFlo for (A) tdTomato fluorescence. (B) The sorted tdTomato + cells were plated into a soft agar assay alongside CMV-TdTomato transduced pMEC. (PDF 1741 kb)
Additional file 8: Figure S8. Representative images depicting differential *in vitro* morphology of populations of pMEC transduced by PGK-Tag-CMV-tdTomato lentivirus. Red fluorescence (RFP) is included to illustrate the percentage of transduced cells. (A) Transduced CD140a-CD49f + pMEC retained a cobblestone morphology characteristic of MEC. (B) Transduced CD140a-tdTomato + pMEC from pig 27-3 grew as elongated cells. (C) Transduced CD140- pMEC from pig 28-3 developed foci *in vitro* rather than as a monolayer. (D) Transduced CD140-tdTomato + pMEC from pig 28-6 grew as sheets of cobblestone epithelium with few foci. (PDF 1137 kb)
Additional file 9: Figure S9. Bright field and red fluorescence (RFP) in a xenograft subcutaneous tumor excised from a mouse carrying CD140-CD49+ PGK-Tag-CMV-tdTomato pMEC (ss020513_1) in hydrogel (A) or Matrigel (B). Scale bar = 1 mm. (C) Bright field and RFP of a CD140- PGK-Tag-CMV-tdTomato tumor (ss082112) in Matrigel. Scale bar = 1 mm. Bright field and RFP of microscopic growths in mammary fat pads injected with CD140- pMEC transduced with PGK-Tag-CMV-tdTomato co-injected with (D) Matrigel (ss082112) or (E) hydrogel. Scale bar = 500 μm. (PDF 1166 kb)

## Abbreviations

CMV: cytomegalovirus
EF1α: human elongation factor 1α
FBS: fetal bovine serum
H&E: hematoxylin and eosin
LT: large t antigen
MT: middle t antigen
NSG: *NOD gamma scid* mice (NOD.Cg-*Prkdc*^*scid*^ *Il2rg*^*tm1Wjl*^/SzJ)
PGK: phosphoglycerate kinase
pMEC: porcine mammary epithelial cells
ST: small t antigen
Tag: T antigen
TDLU: terminal ductal lobular unit
