# Supplementary figures and images for: Neoplastic transformation of porcine mammary epithelial cells in vitro and tumor formation in vivo
Source: BMC Cancer. 2015 Jul 31;15:562. doi: 10.1186/s12885-015-1572-7 (PMC4520266; doi:10.1186/s12885-015-1572-7)

Figure S1

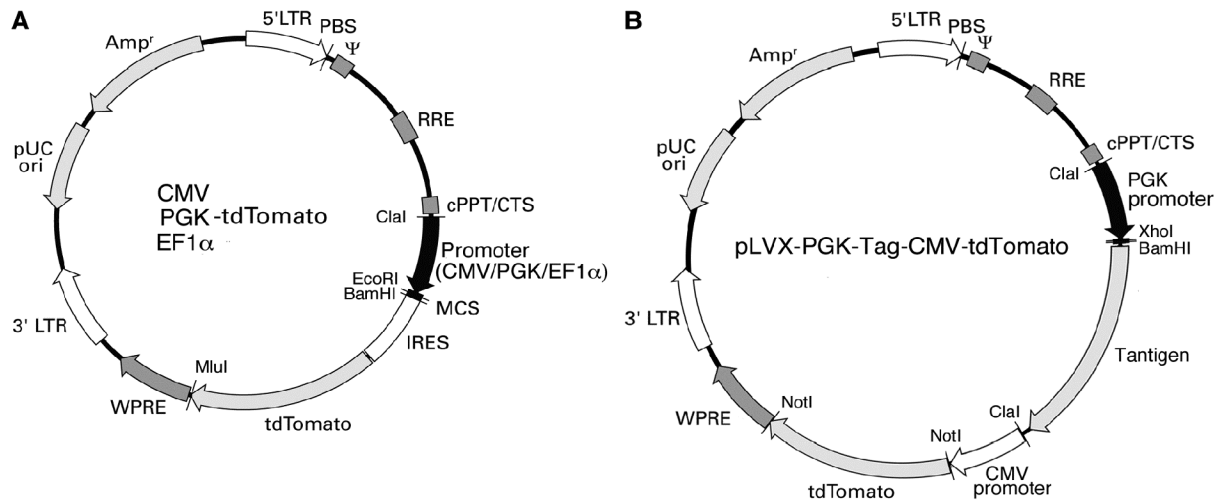

Supplement: Additional file 1: Figure S1. — Plasmid maps of lentiviral vectors. (A) Map of the construct pLVX-IRES-tdTomato (CMV-tdTomato) wherein the CMV promoter directs tdTomato expression. The CMV promoter was replaced by the EF1α or PGK promoter to create EF1α-tdTomato or PGK-tdTomato. (B) To generate the pLVX-PGK-Tag-CMV-tdTomato vector, the pLVX-PGK-Tag vector was first created by replacing the IRES and tdTomato from the vector PGK-tdTomato with murine polyomavirus T antigen that encodes small T (ST), middle T (MT) and large T (LT) antigens. The CMV promoter and tdTomato coding sequence were sequentially added to the vector, creating a pLVX-PGK-Tag-CMV interim vector and the final expression vector pLVX-PGK-Tag-CMV-tdTomato. (PDF 155 kb) [file 12885_2015_1572_MOESM1_ESM.pdf]

Figure S2

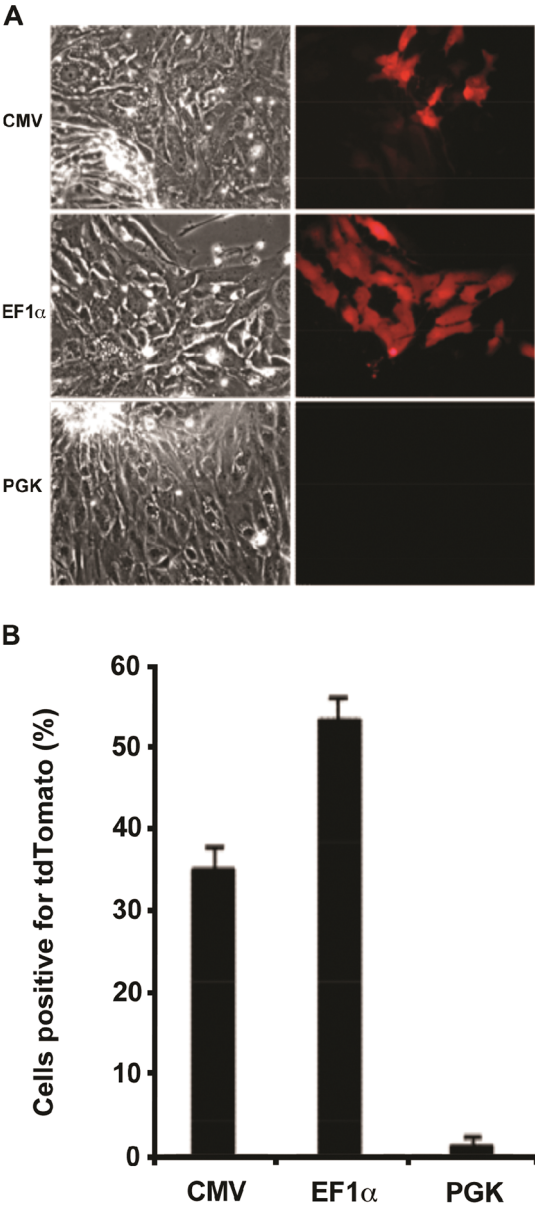

Supplement: Additional file 2: Figure S2. — Transduction of pMEC by CMV-, EF1α- and PGK-TdTomato lentivirus. (A) Representative bright field and corresponding fluorescent images of primary pMEC at passage 0, 7d after transduction by CMV-tdTomato, EF1α-tdTomato or PGK-tdTomato lentiviral constructs. (B) Quantification of cells expressing tdTomato, as a measure CMV, EF1α and PGK promoter activity in pMEC. (PDF 1353 kb) [file 12885_2015_1572_MOESM2_ESM.pdf]

**Figure S3**

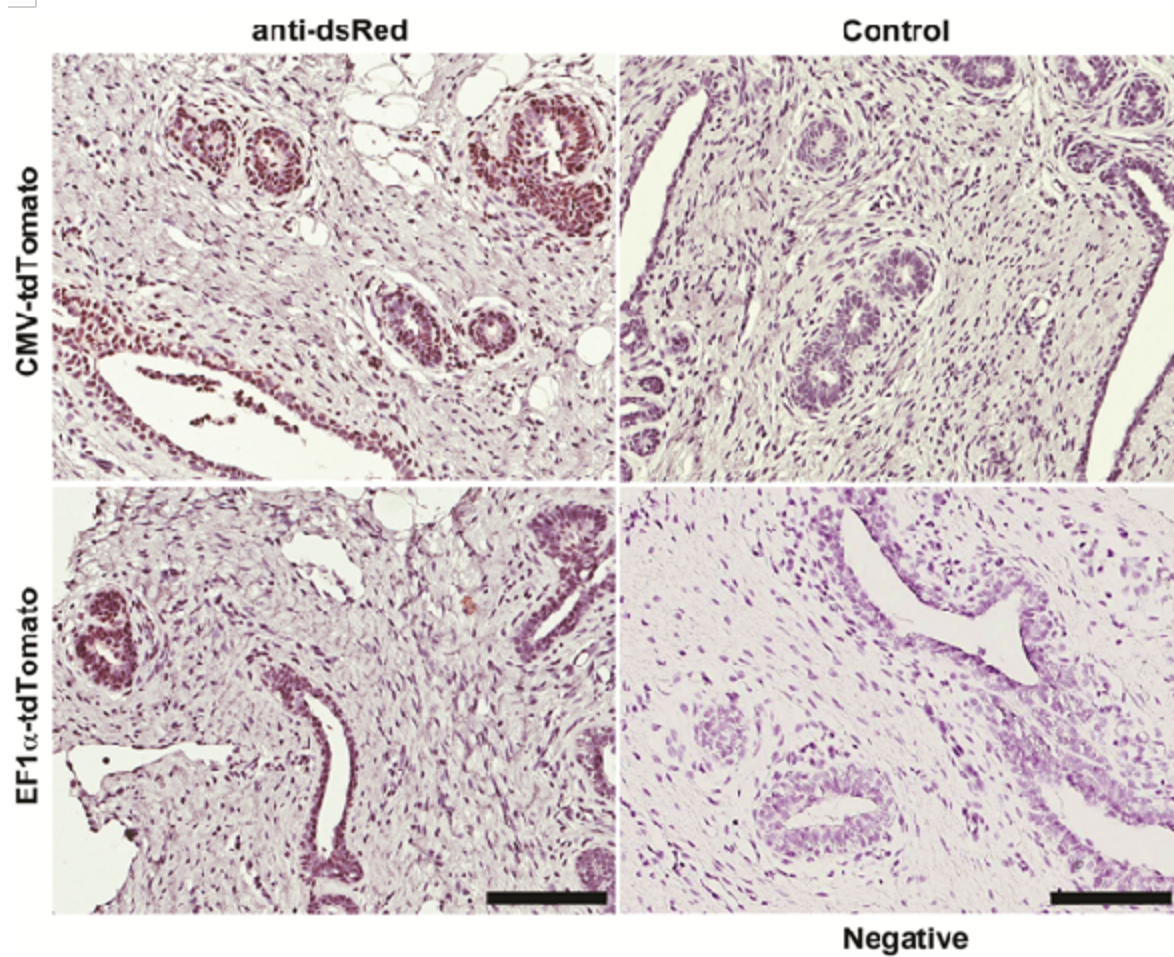

Supplement: Additional file 3: Figure S3. — Lentiviral integration following intraductal injection of lentivirus encoding CMV-tdTomato, EF1α-tdTomato or PGK-tdTomato (n = 9 pigs/lentiviral construct). Mammary tissues were harvested 5d post-injection, processed into paraffin and sections analyzed by immunohistochemical detection of dsRED (monomer of tdTomato) with using NovaRed for detection and a hematoxylin counterstain. The control section was not exposed to the dsRED antibody. The negative section is from a gland that was not injected with tdTomato expressing lentivirus. Scale bar = 100 μm. (PDF 624 kb) [file 12885_2015_1572_MOESM3_ESM.pdf]

Figure S4

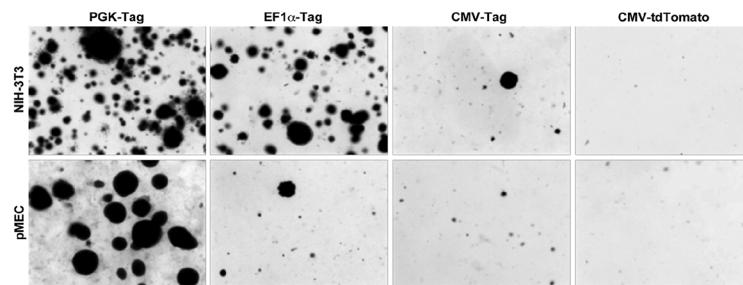

Supplement: Additional file 4: Figure S4. — The promoter of large, middle and small T antigen (Tag) expression affected the rate of anchorage-independent colony formation. Representative images from three replicate experiments to measure soft-agar colony formation by pMEC (n = 2 wells/construct) transduced with CMV-Tag, EF1α-Tag, PGK-Tag or CMV-tdTomato lentivirus (MOI of 100). NIH-3 T3 cells were transduced with the same constructs as a positive control. Cells transduced by CMV-tdTomato served as negative control. (PDF 508 kb) [file 12885_2015_1572_MOESM4_ESM.pdf]

Figure S5

A

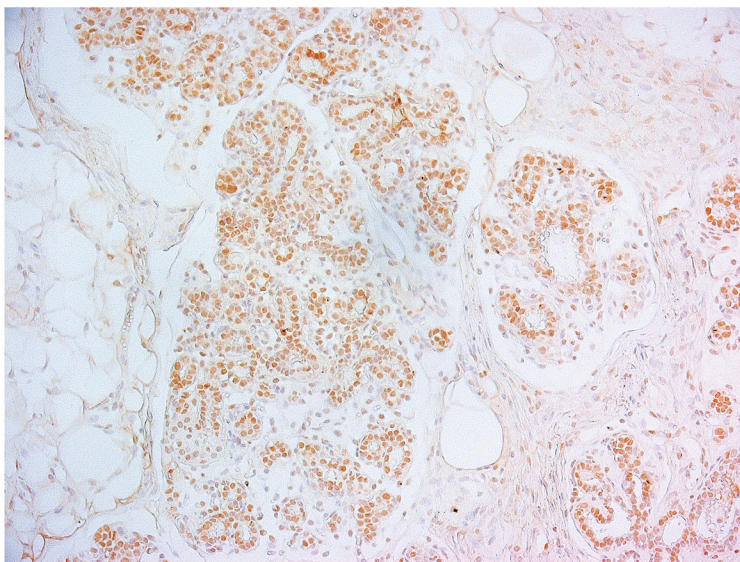

B

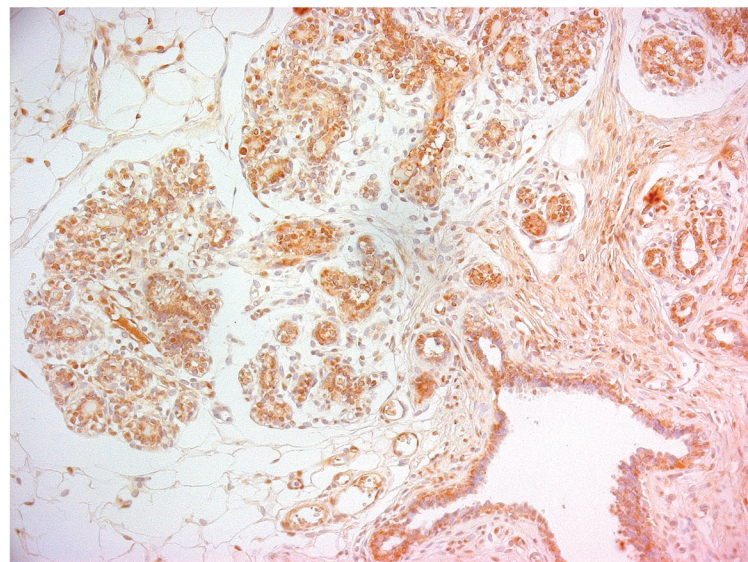

C

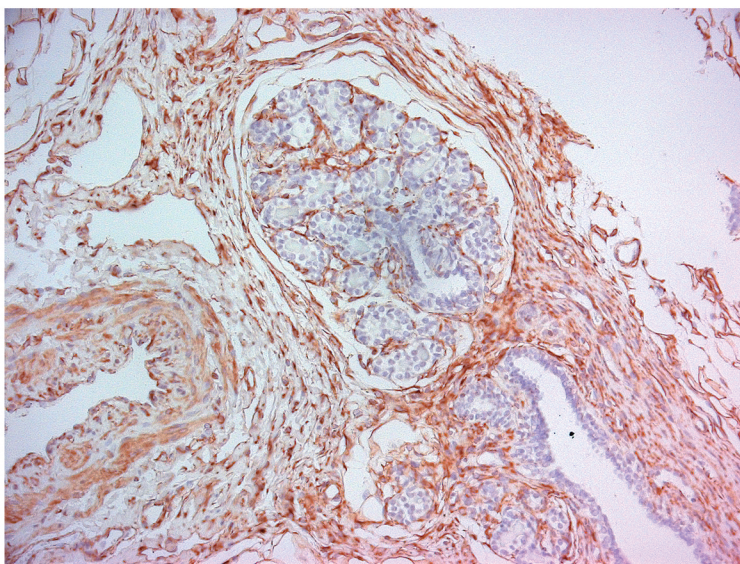

D

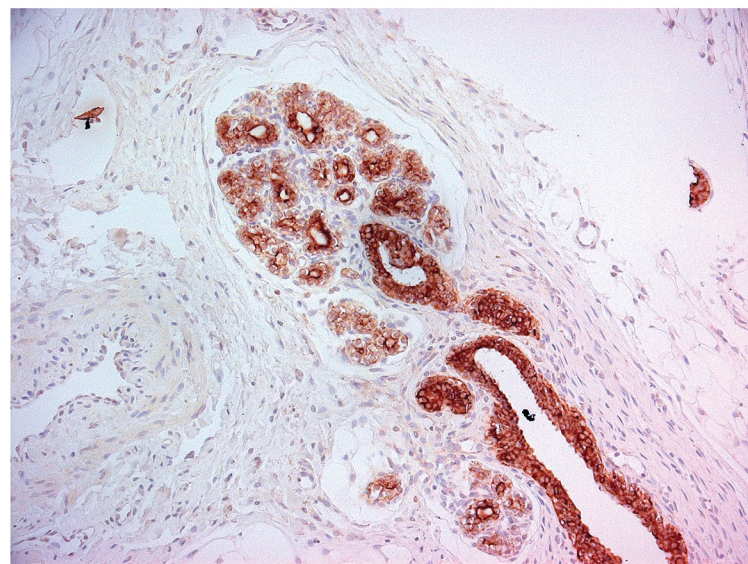

Supplement: Additional file 5: Figure S5. — Immunohistological features of the dense epithelial structures that appeared after isotopic engraftment of PGK-Tag transformed pMEC. Representative images detailing the expression of (A) estrogen receptor (B) progesterone receptor (C) vimentin and (D) cytokeratin. Scale bar = 100 μm. (PDF 9416 kb) [file 12885_2015_1572_MOESM5_ESM.pdf]

Figure S6

A

Cytokeratin

Vimentin

Merged

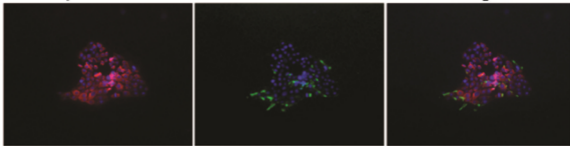

B

P2

P3

P5

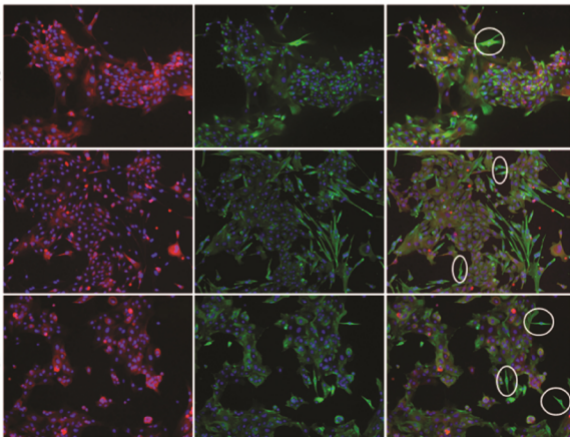

Supplement: Additional file 6: Figure S6. — Vimentin and cytokeratin expression in CD49f-/+ populations. Representative images depicting cytokeratin (red) and vimentin (green) expression in FACS sorted pMEC. Cells affixed to glass slides were stained by immunofluorescence for both pan-cytokeratin and vimentin. (A) CD140-CD49- pMEC. (B) CD140a-CD49f + pMEC at passages 2 (P2), 3 (P3) and 5 (P5). Vimentin-only positive cells are circled. (PDF 846 kb) [file 12885_2015_1572_MOESM6_ESM.pdf]

**A**

Bright Field

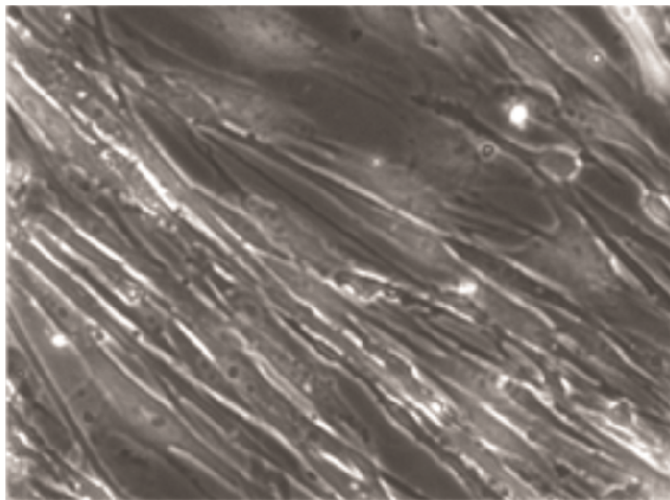

RFP

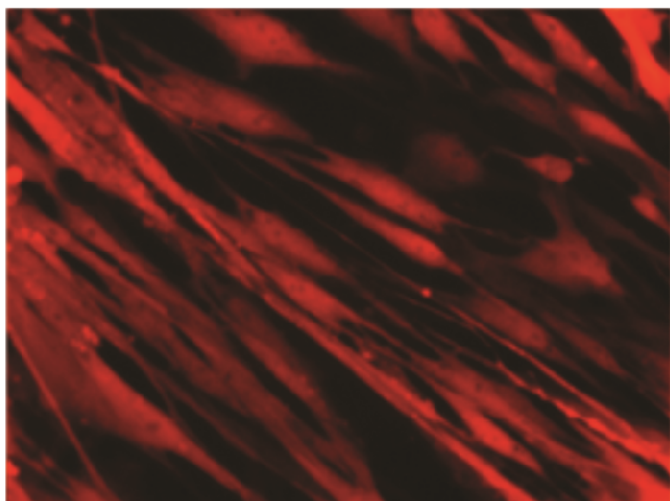

Merged

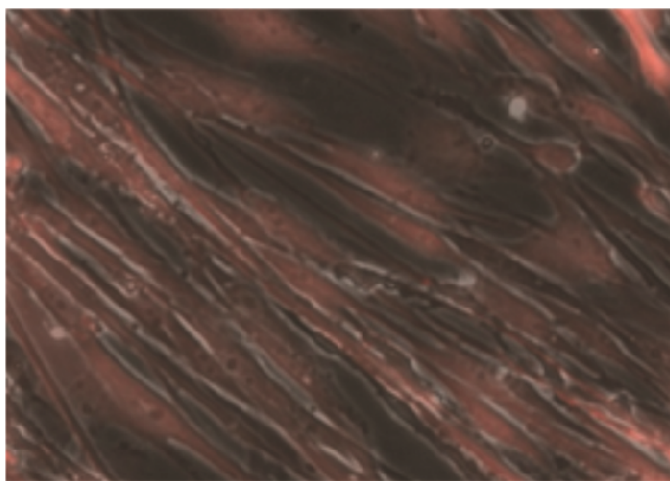

**B**

pMEC\_PGK-Tag-CMV-tdT

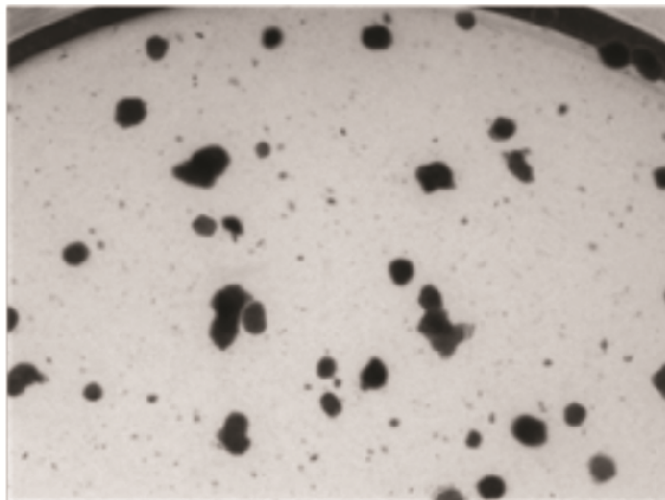

pMEC\_CMV-tdTomato

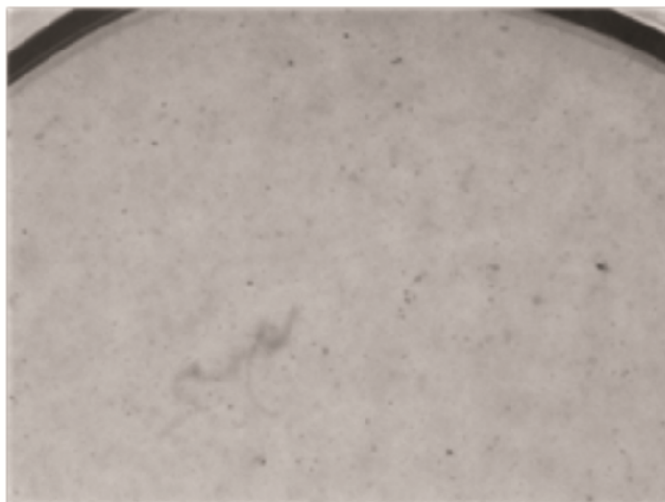

Supplement: Additional file 7: Figure S7. — Porcine mammary epithelial cells (pMEC) were transduced with PGK-Tag-CMV-TdTomato (PGK-Tag-CMV-tdT) lentivirus, cultured for 7d then sorted using a MoFlo for (A) tdTomato fluorescence. (B) The sorted tdTomato + cells were plated into a soft agar assay alongside CMV-TdTomato transduced pMEC. (PDF 1741 kb) [file 12885_2015_1572_MOESM7_ESM.pdf]

Figure S8

**BRIGHT FIELD**

**RFP**

**MERGE**

**A**

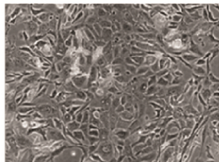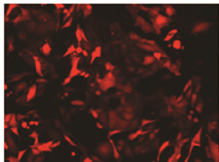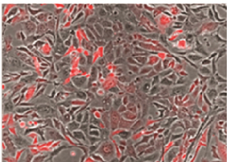

**B**

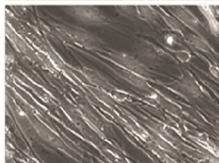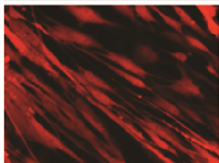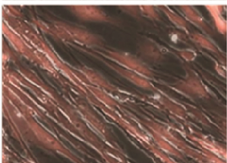

**C**

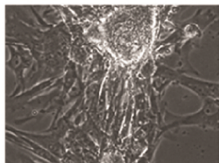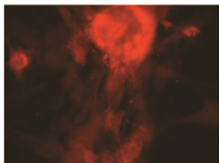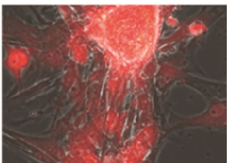

**D**

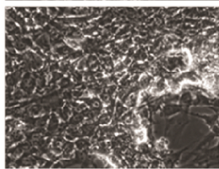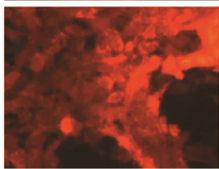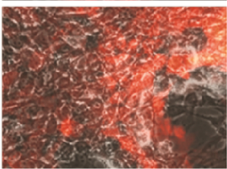

Supplement: Additional file 8: Figure S8. — Representative images depicting differential in vitro morphology of populations of pMEC transduced by PGK-Tag-CMV-tdTomato lentivirus. Red fluorescence (RFP) is included to illustrate the percentage of transduced cells. (A) Transduced CD140a-CD49f + pMEC retained a cobblestone morphology characteristic of MEC. (B) Transduced CD140a-tdTomato + pMEC from pig 27-3 grew as elongated cells. (C) Transduced CD140- pMEC from pig 28-3 developed foci in vitro rather than as a monolayer. (D) Transduced CD140-tdTomato + pMEC from pig 28-6 grew as sheets of cobblestone epithelium with few foci. (PDF 1137 kb) [file 12885_2015_1572_MOESM8_ESM.pdf]

Figure S9

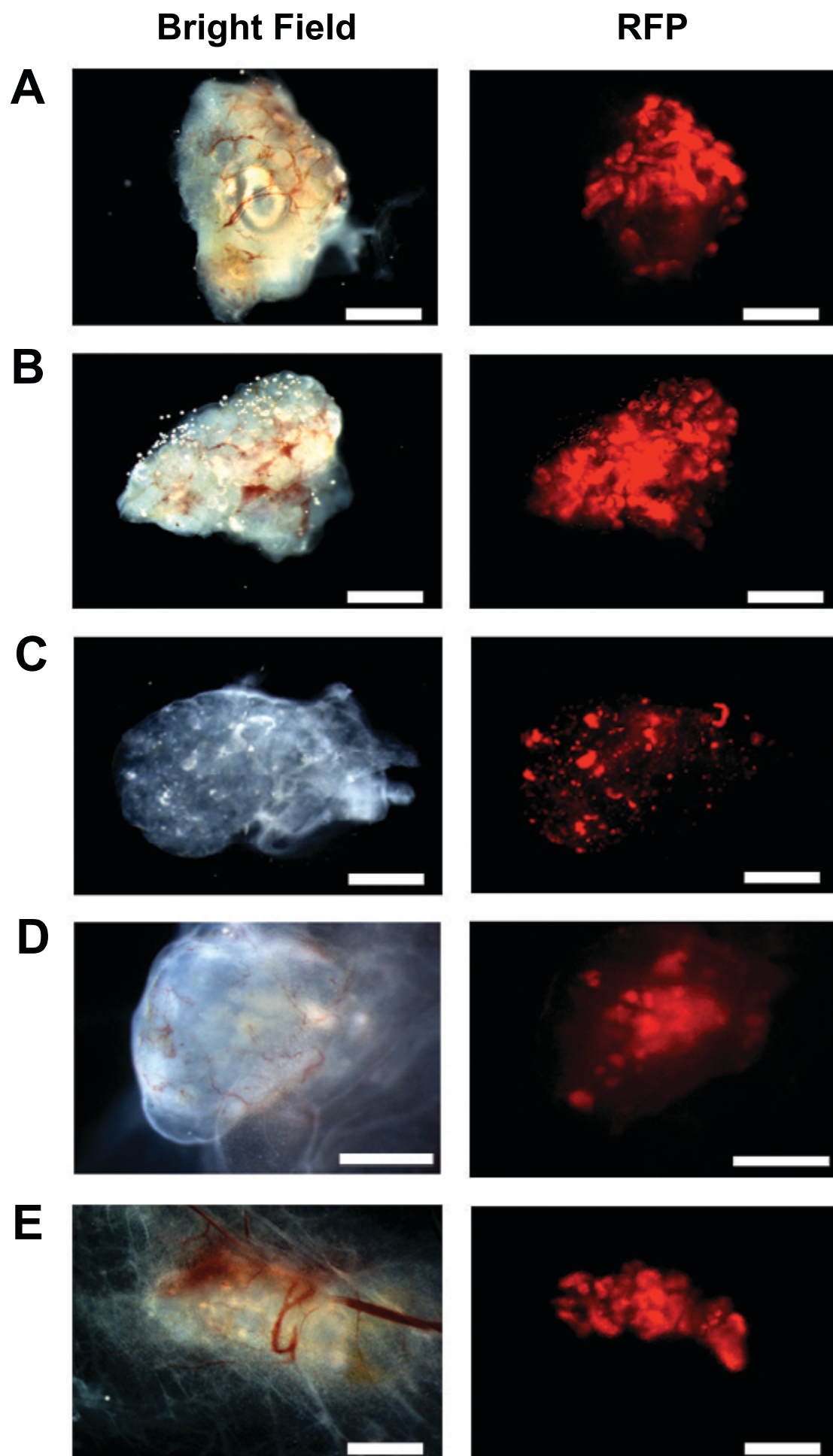

Supplement: Additional file 9: Figure S9. — Bright field and red fluorescence (RFP) in a xenograft subcutaneous tumor excised from a mouse carrying CD140-CD49+ PGK-Tag-CMV-tdTomato pMEC (ss020513_1) in hydrogel (A) or Matrigel (B). Scale bar = 1 mm. (C) Bright field and RFP of a CD140- PGK-Tag-CMV-tdTomato tumor (ss082112) in Matrigel. Scale bar = 1 mm. Bright field and RFP of microscopic growths in mammary fat pads injected with CD140- pMEC transduced with PGK-Tag-CMV-tdTomato co-injected with (D) Matrigel (ss082112) or (E) hydrogel. Scale bar = 500 μm. (PDF 1166 kb) [file 12885_2015_1572_MOESM9_ESM.pdf]
